# Electrophilically Activated Nitroalkanes in Synthesis of 3,4-Dihydroquinozalines

**DOI:** 10.3390/molecules26144274

**Published:** 2021-07-14

**Authors:** Alexander V. Aksenov, Igor Yu. Grishin, Nicolai A. Aksenov, Vladimir V. Malyuga, Dmitrii A. Aksenov, Mezvah A. Nobi, Michael Rubin

**Affiliations:** 1 Department of Chemistry, North Caucasus Federal University, 1a Pushkin St., 355017 Stavropol, Russia; igrishin@ncfu.ru (I.Y.G.); naksenov@ncfu.ru (N.A.A.); vmaliuga@ncfu.ru (V.V.M.); daksenov@ncfu.ru (D.A.A.); 2 Department of Chemistry, University of Kansas, 1567 Irving Hill Road, Lawrence, KS 66045, USA; mezvah_nobi@ku.edu

**Keywords:** nitroalkanes, heterocyclic compounds, quinozalines, annulation

## Abstract

Nitroalkanes activated with polyphosphoric acid serve as efficient electrophiles in reactions with various nucleophilic amines. Strategically placed second functionality allows for the design of annulation reactions enabling preparation of various heterocycles. This strategy was employed to develop an innovative synthetic approach towards 3,4-dihydroquinazolines from readily available 2-(aminomethyl)anilines.

## 1. Introduction

**Figure 1:**
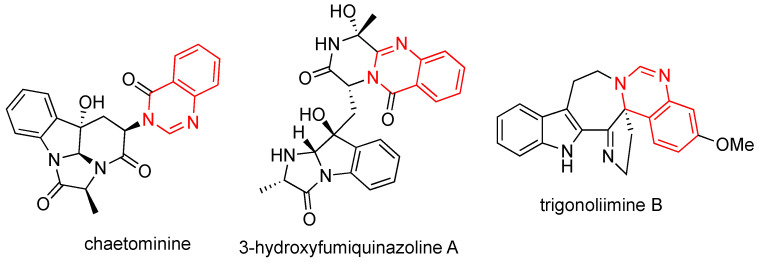
Biologically active natural 3,4-dihydroquinazolines.

3,4-Dihydroquinazolines belong to privileged scaffolds widely used in drug design, with their importance in medicinal chemistry being difficult to overstate. This structural fragment is found in thousands of natural products and synthetic medicinal agents, including many alkaloids with valuable biological activities. Among them are cytotoxic alkaloid chaetominine isolated from endophytic fungi of the *Chaetomium* genus [1] and 3-hydroxyfumiquinazoline A isolated from the fungus *Aspergillus fumigatus*, which has demonstrated promising antifungal and insecticidal activities [2] (Figure 1). Additionally, a family of trigonoliimine alkaloids were isolated from the leaves of tropical plants from the genus *Trigonostemon* [3]. Due to their highly unusual polycyclic scaffolds and newly discovered anti-cancer and antiviral activities, these trigoliimines are in focus of multiple synthetic exercises [4,5,6,7,8]. Still, elaboration of novel synthetic methods allowing for the efficient assembly of the 3,4-dihydroquinazoline core is exceedingly desired. Herein, we wish to disclose an account of our recent progress towards preparation of these important heterocycles via annulation involving electrophilically activated nitroalkanes. 

## 2. Results and Discussion

**Scheme 1:**
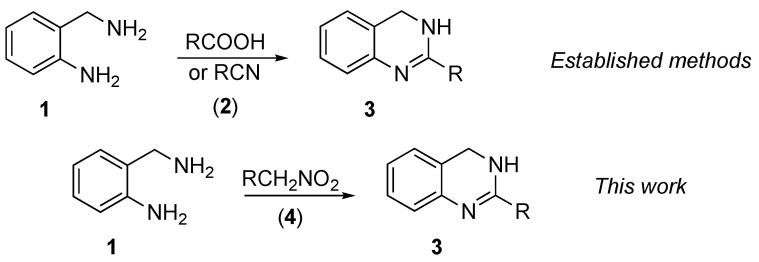
Annulations of 2-(aminomethyl)anilines to afford 3,4-dihydroquinazolines.

Annulation of 2-(aminomethyl)anilines to afford a 3,4-dihydroquinozaline core is one of the most common reactions, returning almost 600,000 matches in a SciFinder database search. Since 2-(aminomethyl)aniline (**1**) can be viewed as a bis-nucleophilic synthon, all these annulations are designed as double-fold attacks at reagents that can serve as a 1,1-bis-electrophilic synthetic equivalent (Scheme 1). Typically, carboxylic acids [9,10,11,12,13], nitriles [14], or other related carbonyl derivatives [15,16,17] (**2**) are employed (Scheme 1). Oxidative protocols utilizing aldehydes as alkylating reagents were also reported [18,19,20]. Lately, our research group was instrumental in designing novel multistep cascade transformations employing nitroalkanes as electrophilic or bis-electrophilic synthons. 

**Scheme 2:**
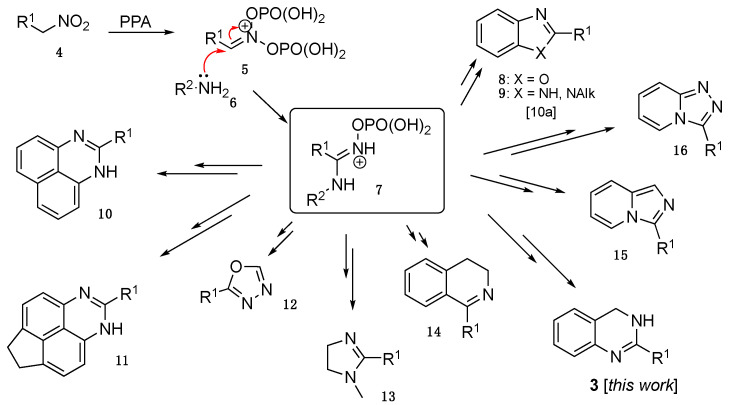
Synthesis of nitrogen-based heterocycles employing electrophilically activated nitroalkanes.

Although nitroalkanes (**4**) normally behave as pronucleophilic species; in a polyphosphoric acid medium they are stabilized in a form of phosphorylated nitronates (**5**) with profound electrophilic properties. These phosphorylated nitronates tend to react with various carbon-based nucleophiles, for instance electron-rich arenes, allowing for Friedel–Crafts-type C–H functionalization reactions, often with subsequent accompanying rearrangements [21,22,23,24]. Furthermore, it was found that nitrogen-based nucleophiles (**6**), such as aliphatic amines [25,26,27], anilines [28,29,30,31], or hydrazines [32,33], can be utilized instead of water in a process mechanistically related to the classical Nef reaction [34,35,36]. Employing strategically placed second nucleophilic functionality (carbon or heteroatom-based), the resulting amidinium species (**7**) can be transformed into a variety of heterocyclic compounds, such as benzoxazoles (**8**) [23,28] or benzimidazoles (**9**) [28], perimidines (**10**) [29], 6,7-dihydro-1*H*-cyclopenta[gh]perimidines (**11**) [31], 1,3,4-oxa-di-azoles (**12**) [32,33], imidazolines (**13**) [25], 3,4-dihydro-iso-quinolines (**14**) [27], imidazo [1,5-a]pyridines (**15**) [26], and [1,2,4]triazolo[4,3-a]pyridines (**16**) [37] (Scheme 2). 

**Scheme 3:**
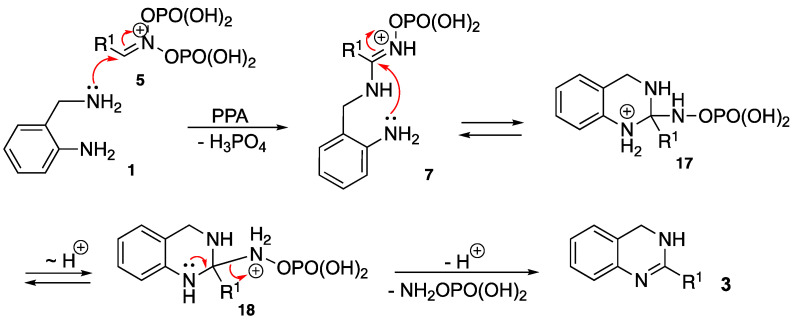
Mechanistic rationale of the featured annulation.

It occurred to us that a similar strategy can be applied for the straightforward preparation of 3,4-dihydroquinazolines as well. In reaction with bis-nucleophilic 2-(aminomethyl)anilines (**1**), the phosphorylated nitronate species **5** would, after elimination of orthophosphoric acid entity, afford an amidinium intermediate **7** (Scheme 3). The latter would then experience intramolecular 6-*exo-trig* nucleophilic attack of the aniline group to the iminium moiety to furnish heterocyclic ring **17**. Subsequent proton transfer to the exocyclic nitrogen atom would provide intermediate **18**, which after elimination of hydroxylamine phosphate would afford the desired product **3** (Scheme 3).

With this idea in mind, we attempted to carry out reactions between 2-(1-amino ethyl)aniline (**1a**) and 1-nitropropane (**4c**) in a molar ratio of 1:2. In the initial experiment, a mixture of these reagents was heated in 86% orthophosphoric acid at 110 °C (Table 1, entry 1) or 120 °C (entry 2). However, the reaction did not proceed under these conditions. Next, a mixture of 86% orthophosphoric acid and 87% polyphosphoric acids (approximately corresponding to anhydrous H_3_PO_4_) or molten 87% polyphosphoric acid (PPA) were employed as the media for the reactions carried out at 120 °C. Again, the reactions did not proceed, and the starting amine **1a** remained intact in both cases (entries 3, 4). As it was shown in our previous report [25] that addition of phosphorous acid to the reaction medium could enable stubborn steric-restricted annulations, we also decided to attempt to stimulate the reactivity using this trick. To this end, the reaction of **1a** and **4c** was carried out in a mixture of H_3_PO_3_ and 87% PPA (1:1 m/m) at 130 °C. Formation of product **3ac** was detected, albeit in a low yield (entry 5). To improve the reaction performance, we attempted to increase the temperature. At 150 °C, the reaction outcome still remained quite marginal (entry 6). However, at 160 °C, a synthetically meaningful result was obtained (entry 7). We also tested the reaction in pure phosphorous acid without addition of PPA. This reaction was accompanied with partial thermal decomposition of the medium, which complicated the isolation and rendered notably lower yield (entry 8).

**Scheme 4:**
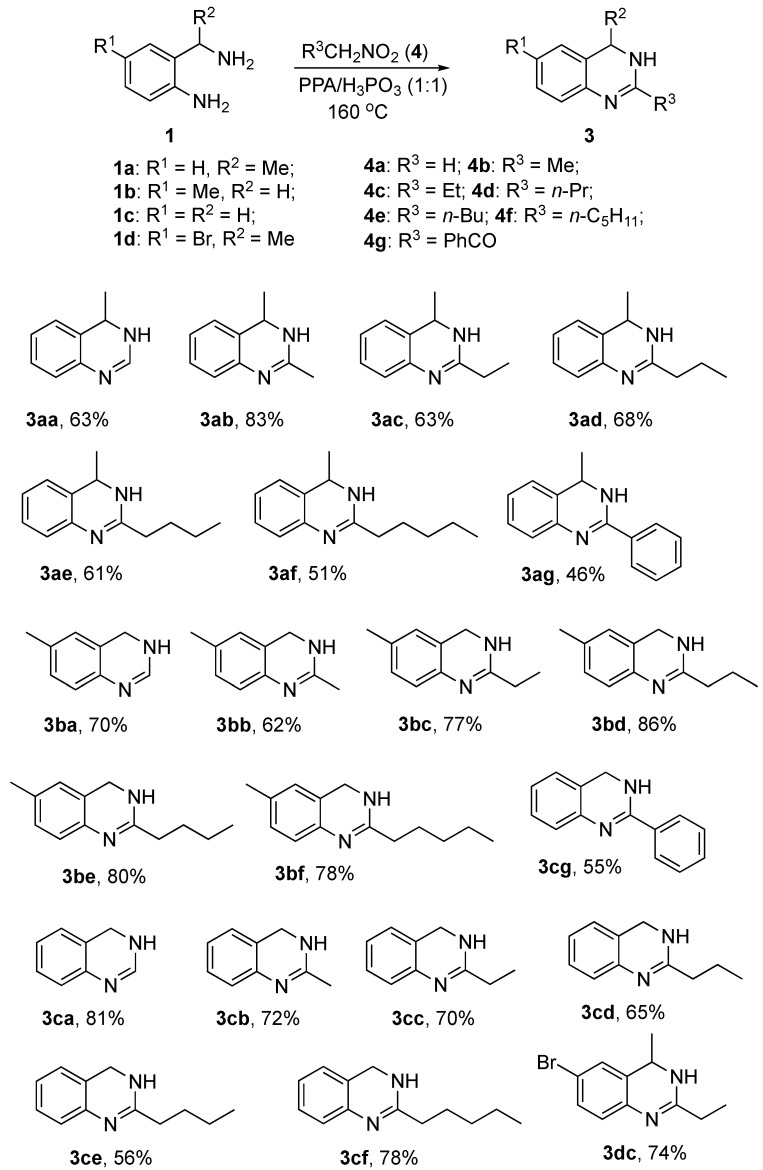
Examples of 3,4-dihydroquinazolines obtained in annulation of 2-(aminomethyl)anilines and nitroalkanes. Isolated yield of purified products is listed.

With optimized conditions in hand, we then proceeded to investigate the scope of the featured transformation. The results presented in Scheme 4 demonstrate that 3,4-dihydroquinazolines substituted with alkyl groups at C-2, C-4, or C-6 can be routinely obtained via this method in good to excellent yields. It should be pointed out that alkyl substituents at these positions as well as the length of the alkyl chain in the nitroalkane reagent do not seem to affect the efficiency of the featured transformation. Aromatic halide substituent in substrate 1d was also well tolerated, allowing for efficient preparation of brominated product **3cd** (Scheme 4). 

**Scheme 5:**
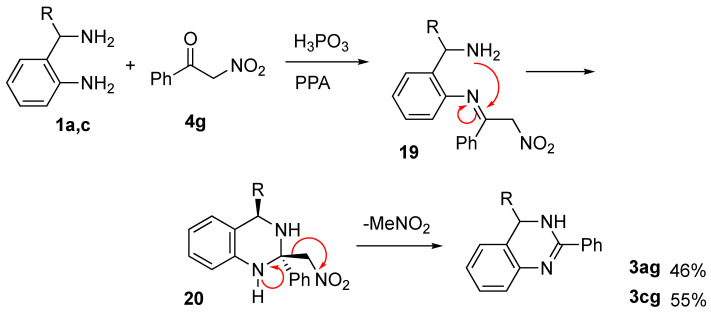
Mechanistic rationale of the annulation involving α-nitroacetophenone.

An attempt to use ethyl 2-nitroacetate as an electrophilic component in the described transformation proved unfruitful. The reaction with **1a** produced complex mixtures of unidentified products, and none of them was isolated in a meaningful yield. More interesting results were obtained in reactions employing α-nitroacetophenone (**4g**). The products of these reactions, **3ag** and **3cg**, were identified as 3,4-dihydroquinozalines bearing phenyl substituents at C-2 (Scheme 4 and Scheme 5). It is believed that the mechanism of this transformation involves initial formation of imine **19** via condensation of ketone **4g** with one of the available amino groups. Subsequent 6-*endo*-*trig* nucleophilic cyclization took place, providing cyclic aminal **20**, which underwent further elimination of nitromethane moiety to afford the observed products (Scheme 5). 

## 3. Materials and Methods

General. NMR spectra, ^1^H and ^13^C were measured in solutions of CDCl_3_ or DMSO-*d*_6_ on a Bruker AVANCE-III HD instrument (at 400.40 or 100.61 MHz, respectively). Residual solvent signals were used as internal standards, in DMSO-*d*_6_ (2.50 ppm for ^1^H, and 40.45 ppm for ^13^C nuclei) or in CDCl_3_ (7.26 ppm for ^1^H, and 77.16 ppm for ^13^C nuclei). Signals in ^13^C NMR spectra marked with an asterisk (*) were assigned based on 2D ^1^H-^13^C HMBC and ^1^H-^13^C HSQC experiments. HRMS spectra were measured on a Bruker maXis impact (electrospray ionization, in MeCN solutions, employing HCO_2_Na-HCO_2_H for calibration). IR spectra were measured on an FT-IR spectrometer Shimadzu IRAffinity-1S equipped with an ATR sampling module. The reaction progress, the purity of isolated compounds, and the R*_f_* values were monitored with TLC on Silufol UV-254 plates. Column chromatography was performed on silica gel (32–63 μm, 60 Å pore size). Melting points were measured with a Stuart SMP30 apparatus. Polyphosphoric acid (87%) was prepared by dissolving a precisely measured amount of P_2_O_5_ in 85% orthophosphoric acid. All the reagents and solvents were purchased from commercial venders and used as received.

*2-(1-Aminoethyl)aniline* (**1a**):

This compound was obtained via transformation of acetophenone into the corresponding oxime and its subsequent reduction with either sodium borohydride in the presence of TiCl_4_ in dimethoxyethane (DME) (method A), or with zinc dust in hydrochloric acid (method B). 

Accordingly, 1-(2-nitrophenyl)ethan-1-one oxime was obtained via published procedure [38] by addition of hydroxylamine hydrochloride (4.45 g, 64.0 mmol) and sodium acetate (6.56 g, 80 mmol) to a solution of 2-nitroacetophenone (6.61 g, 40.0 mmol) in an ethanol and water mixture (4:1, 100 mL). The reaction mixture was boiled at reflux for 2 h (monitored with TLC), and cooled to RT. Then a third of the solvent volume was removed in vacuum, and the residual solution was poured into 100 mL of crushed ice and water mixture. Formed white flaky precipitate was filtered and dried in air. Yield: 6.45 g (90%). Colorless crystalline mass, mp 92–95 °C (Literature data: mp 91 °C [39], mp 115.5–116.5 °C (aq. methanol) [40].

The oxime was subsequently reduced via one of the following procedures:

Method A:

A solution of 1-(2-nitrophenyl)ethan-1-one oxime (1.80 g, 10.0 mmol) in anhydrous DME was added slowly under argon atmosphere to a stirred and chilled in ice-bath mixture of sodium borohydride (1.59 g, 42 mmol) and titanium (IV) chloride (3.99 g, 21 mmol) in anhydrous DME (40 mL), and the mixture was stirred at room temperature for 20 h [41]. Then, the reaction mixture was carefully poured into ice-cold water (100 mL), neutralized with aqueous ammonia (25%), and concentrated in vacuum. The residual solid was extracted in a Soxhlet apparatus for 8 h into isopropyl alcohol (230 mL). The extract was concentrated in vacuum to obtain crude 2-(1-aminoethyl)aniline chlorohydride as off-white powder, mp 172–179 °C (Literature data: mp 187 °C (decomp) [42]. Yield: 1.58 g (9.1 mmol, 91%). For isolation of 2-(1-aminoethyl)aniline as free base, the salt was dissolved in water, washed with ethyl acetate, basified with aqueous NaOH (20%), and extracted with ethyl acetate. Combine organic extracts were washed with brine, dried with sodium sulphate, and concentrated in vacuum.

Method B:

To a solution of 1-(2-nitrophenyl)ethan-1-one oxime (2.07 g, 11.5 mmol) in a mixture of ethanol (45 mL), water (12 mL) and concentrated HCl (24 mL) stirred at 0 °C zinc duct was added in small portions over 30 min (9.03 g, 138.1 mmol) [43]. The reaction mixture was stirred at 50 °C for 1.5 h (the reaction progress was monitored with TLC), then cooled down to room temperature, and filtered. The filtrate was concentrated in vacuum, and the residue was basified with aqueous ammonia (25%) to pH 9 and extracted with ethyl acetate (3 × 50 mL). Combined organic extracts were dried with sodium sulphate and concentrated in vacuum. The residual amber-colored oil was dissolved in diluted aqueous HCl (70 mL, pH of resulting solution c.a. 4–5), and washed with ethyl acetate (3 × 25 mL). The aqueous phase was basified with ammonia (25%) to pH 9 and extracted with ethyl acetate (3 × 25 mL). Combined organic phases were washed with brine, dried with sodium sulphate, and concentrated. 2-(1-Aminoethyl)aniline was obtained as pale-yellow oil, slowly solidifying upon standing. Grinding of this crystalline mass afforded pale-yellow powder, mp 85–89 °C. Yield: 1.19 g (8.4 mmol, 73%). ^1^H NMR (400 MHz, DMSO-*d*_6_) δ 7.04 (dd, *J* = 7.5, 1.6 Hz, 1H), 6.88 (td, *J* = 7.6, 1.6 Hz, 1H), 6.56 (dd, *J* = 7.9, 1.3 Hz, 1H), 6.48 (td, *J* = 7.4, 1.3 Hz, 1H), 4.03 (q, *J* = 6.6 Hz, 1H), 1.26 (d, *J* = 6.6 Hz, 3H).

*2-(Aminomethyl)-4-methylaniline* (**1b**):

This compound was obtained starting from *p*-toluidine via iodination and subsequent transformation of the resulting 2-iodo-4-methylaniline into 2-amino-5-methylbenzonitrile, which was reduced with lithium aluminum hydride in tetrahydrofuran (THF).

Accordingly, 2-iodo-4-methylaniline was obtained according to the known procedure [44,45]. To this end, a mixture of *p*-toluidine (2.65 g, 24.7 mmol), iodine (6.15 g, 24.2 mmol), and sodium bicarbonate (2.70 g, 32.1 mmol), methylene chloride (50 mL) and water (25 mL) was stirred at room temperature for 14 h. The organic phase was separated, and the aqueous layer was extracted with methylene chloride (3 × 10 mL). Combined organic extracts were washed with brine (20 mL) and dried with anhydrous sodium sulphate. Solvent was evaporated in vacuum, and the residue was purified by preparative column chromatography eluting with a mixture of ethyl acetate and light petroleum ether (1:20). 2-Iodo-4-methylaniline was obtained as yellow oil, *R_f_* 0.47 (EtOAc/hexane, 1:15). Yield: 5.03 g (21.1 mmol, 87%). This material was used for the following step without additional purification.

2-Amino-5-methylbenzonitrile was synthesized according to the known procedure [46]. To a stirred solution of 2-iodo-4-methylaniline (4.80 g, 20.5 mmol) in DMF (25 mL) was added copper (I) cyanide (2.75 g, 30.8 mmol). The reaction mass was stirred and heated at 130 °C for 3 h, then cooled to room temperature. Aqueous sodium cyanide (100 mL of 10% solution) was added (caution, toxic) and the mixture was stirred for an additional 1 h. The aqueous phase was extracted with ethyl acetate (3 × 40 mL). Combined organic extracts were washed with brine, dried with sodium sulphate, and concentrated in vacuum. The residual crude oil was purified by preparative column chromatography on silica gel eluting with a mixture of ethyl acetate and light petroleum ether (1:5). 2-Amino-5-methylbenzonitrile was obtained as light-caramel crystals, mp 59–61 °C (Literature data: mp 60.5–61 °C [47]), *R_f_* 0.66 (EtOAc/hexane, 1:2). Yield: 2.11 g (16.0 mmol, 78%). 

Finally, 2-(aminomethyl)-4-methylaniline (**1b**) was obtained according to the known method [48], replacing borane with lithium aluminum hydride (mole ratio LiAlH_4_/nitrile 4:1). To this end, to a stirred under argon atmosphere solution of LiAlH_4_ (2.15 g, 56.7 mmol) in anhydrous THF (20 mL), a solution of 2-amino-5-methylbenzonitrile 1.83 g (13.8 mmol) in dry THF (10 mL) was added dropwise over 30 min at room temperature. The mixture was stirred for 5 h, and the reaction progress was monitored with TLC. After complete consumption of nitrile, water (5 mL) was carefully added dropwise, followed by 20% aqueous sodium hydroxide (2 mL). Formed precipitate was filtered, and the filter cake was washed with several portions of hot chloroform (combined volume 100 mL). Combined organic phases were washed with brine, dried with sodium sulphate and concentrated to obtain the titled compound as light-yellow crystalline powder, mp 72–75 °C. Yield: 1.75 g (10.4 mmol, 93%). ^1^H NMR (400 MHz, Chloroform-*d*) δ 6.96–6.81 (m, 2H), 6.60 (d, *J* = 7.9 Hz, 1H), 3.86 (s, 2H), 2.23 (s, 3H).

*2-(Aminomethyl)aniline* (**1c**):

This compound was obtained analogously to diamine **1a** from 2-nitrobenzaldehyde via its conversion to the corresponding oxime and subsequent reduction either with sodium borohydride in DME in the presence of TiCl_4_ (method A), or with zinc dust in hydrochloric acid (method B). 

2-Nitrobenzaldehyde oxime was obtained in a way similar to preparation of 2-nitroacetophenone oxime described above. To this end, a mixture of 2-nitrobenzaldehyde (6.04 g, 40.0 mmol), hydroxylamine hydrochloride (4.45 g, 64.0 mmol), and sodium acetate (6.56 g, 80 mmol) was boiled in a mixture of ethanol and water (4:1, 100 mL) for 1 h. Crude product was re-crystallized from benzene to afford colorless crystals, mp 99–101 °C. (Literature data, mp 102–103 °C [49]). Yield: 4.98 g (30 mmol, 75%). This material was subjected to the subsequent reduction using one of the following procedures.

Method A:

The reaction was performed in a way similar to the one described above for the preparation of 2-(1-aminoethyl)aniline (**1a**), adding dropwise a solution of 2-nitrobenzaldehyde oxime (1.66 g, 10.0 mmol) in dry DME (10 mL) to a stirred ice-cold mixture of sodium borohydride 1.59 g (42 mmol), titanium (IV) chloride (3.99 g, 21 mmol), and anhydrous DME (40 mL). The mixture was stirred at room temperature for 20 h [43]. After aqueous workup and neutralization with aqueous ammonia (25%), crude material was extracted in Soxhlet apparatus with isopropyl alcohol (210 mL). The extract was concentrated to 2/3 of the initial volume, and the formed precipitate was filtered off to afford 2-(aminomethyl)aniline hydrochloride as off-white flakes, mp 197–199 °C (Literature data: mp 197–199 °C (methanol/diethyl ether) [50]). Yield: 0.75 g (4.8 mmol, 48%).

Transformation of this hydrochloride into free amine was performed by dissolving the salt in water, washing the solution with ethyl acetate, basification of the aqueous phase with aqueous ammonia to pH 9, and extraction with ethyl acetate in the same manner as described above for material **1a**. Combined organic extracts were dried and concentrated in vacuum to afford free titled amine.

Method B:

Reduction with zinc dust was performed according to the typical protocol described above for preparation of **1a** [43]. Zinc dust 9.03 g (138.1 mmol) was added in small portions to a mixture of 2-nitrobenzaldehyde oxime 1.91 g (11.5 mmol), ethanol (45 mL), water (12 mL), and concentrated HCl (24 mL) vigorously stirred at 0 °C. The reaction mixture was stirred for 1.5 h at 50 °C, and the reaction progress was monitored with TLC. The aqueous work up, extraction, and isolation of the product was performed in the same manner as described above in protocol for preparation of compound **1a**. The titled material was afforded as light-yellow crystals, mp 59–60 °C (Literature data: mp 58–59 °C (MeOH, CHCl_3_) [51]). Yield: 0.97 g (7.9 mmol, 69%). ^1^H NMR (400 MHz, Chloroform-*d*) δ 7.14–7.01 (m, 2H), 6.76–6.65 (m, 2H), 3.90 (s, 2H).

*2-(1-Aminoethyl)-4-bromoaniline* (**1d**):

This compound was obtained according to the procedure analogous to those described above for diamines **1a** and **1c** via transformation of 1-(2-amino-5-bromophenyl)ethan-1-one (1.07 g, 5.0 mmol) into the corresponding oxime and its subsequent reduction with zinc dust in hydrochloric acid (method B) according to published literature procedure [41,43]. Overall yield: 0.24 g (1.1 mmol, 22%). Light yellow viscous oil. ^1^H NMR (400 MHz, Chloroform-*d*) δ 7.17 (d, *J* = 2.4 Hz, 1H), 7.13 (dd, *J* = 8.4, 2.4 Hz, 1H), 6.52 (d, *J* = 8.3 Hz, 1H), 4.13 (q, *J* = 6.6 Hz, 1H), 1.45 (d, *J* = 6.6 Hz, 3H). ^13^C NMR (101 MHz, Chloroform-*d*) δ 145.2, 131.3, 130.5, 129.9, 118.0, 109.5, 50.1, 22.7. IR, v_max_/cm^−1^: 3446, 3364, 2969, 1607, 1414, 1281, 1099, 814. HRMS (ES TOF) calculated for (M + H)^+^ C_8_H_12_BrN_2_ 215.0177, found 215.0178 (0.5 ppm).

*Preparation of 3,4-dihydroquinazolines* (**3aa**–**3cf**) *via annulation of 2-aminobenzylamines and 1-nitroalkanes (General procedure):*

A 10 mL round-bottomed flask equipped with reflux condenser and magnetic stirring bar was charged with 2-aminobenzylamine (**1a**–**c**, 1 mmol), 1-nitroalkane (**4a**–**f**, 2.0 mmol), 87% PPA (1 g) and phosphorous acid (1 g). The mixture was vigorously stirred and heated to 160 °C for 2 h monitoring the reaction progress with TLC. Note: in reactions involving nitromethane (**4a**: R^3^ = H) or nitroethane (**4b**: R^3^ = Me) an additional portion of nitroalkane (1.0 mmol) should be added after 1.5 h to compensate for evaporation. After consumption of the starting amine, the reaction mass was cooled down to 80–85 °C, transferred to crushed ice (10–15 g), neutralized with aqueous ammonia (25%) to pH 8, and extracted with ethyl acetate (3 × 25 mL). The combined organic extracts were washed with brine, dried with sodium sulphate, and concentrated in vacuum to obtain the crude material, which can be further purified by preparative column chromatography on silica gel.

*4-Methyl-3,4-dihydroquinazoline* (**3aa**):

Off-white powder, mp 62–65 °C (Literature data: mp 81–83 °C [52]), *R_f_* 0.33 (EtOAc/EtOH/TEA, 32:8:1). Yield: 92 mg (0.63 mmol, 63%). ^1^H NMR (400 MHz, DMSO-*d*_6_) δ 7.06 (q, *J* = 6.8, 6.1 Hz, 2H), 6.98–6.88 (m, 2H), 6.76 (d, *J* = 7.8 Hz, 1H), 4.60 (q, *J* = 6.5 Hz, 1H), 1.28 (d, *J* = 6.5 Hz, 3H). ^13^C NMR (101 MHz, DMSO-*d*_6_) δ 146.4, 140.3*, 127.3, 127.0*, 125.6, 123.4, 120.3*, 47.6, 25.7. IR, v_max_/cm^−1^: 3195, 2974, 1614, 1590, 1479, 1452, 1371, 1250, 759. HRMS (ES TOF) calculated for (M + H)^+^ C_9_H_11_N_2_ 147.0918, found 147.0917 (−1.2 ppm).

*2-Methyl-4-methyl-3,4-dihydroquinazoline* (**3ab**):

Pale-yellow crystals, mp 107–110 °C (Literature data: mp 145–146 °C [52]), *R_f_* 0.62 (EtOAc/EtOH/TEA, 32:8:1). Yield: 133 mg (0.83 mmol, 83%). ^1^H NMR (400 MHz, DMSO-*d*_6_) δ 7.05 (td, *J* = 7.5, 1.8 Hz, 1H), 6.95 (dd, *J* = 7.6, 1.7 Hz, 1H), 6.89 (td, *J* = 7.3, 1.3 Hz, 1H), 6.75 (dd, *J* = 7.8, 1.3 Hz, 1H), 4.59 (q, *J* = 6.4 Hz, 1H), 1.87 (s, 3H), 1.27 (d, *J* = 6.4 Hz, 3H). ^1^H NMR (400 MHz, Chloroform-*d*) δ 7.15 (td, *J* = 7.5, 1.6 Hz, 1H), 7.06–6.90 (m, 3H), 5.22 (s, 1H), 4.75 (q, *J* = 6.5 Hz, 1H), 2.06 (s, 3H), 1.44 (d, *J* = 6.5 Hz, 3H). ^13^C NMR (101 MHz, Chloroform-*d*) δ 154.6, 140.7, 128.1, 125.3, 125.2, 124.4, 121.8, 49.2, 25.9, 22.7. ^13^C NMR (101 MHz, DMSO-*d*_6_) δ 127.3, 125.1, 122.8, 48.4, 25.6, 21.7. IR, v_max_/cm^−1^: 3253, 2973, 1617, 1576, 1484, 1436, 1395, 1274, 752. HRMS (ES TOF) calculated for (M + H)^+^ C_10_H_13_N_2_ 161.1070, found 161.1073 (2.0 ppm).

*2-Ethyl-4-methyl-3,4-dihydroquinazoline* (**3ac**):

Viscous glassy oil of amber color, *R_f_* 0.42 (EtOAc/EtOH/TEA, 32:8:1). Yield: 110 mg (0.63 mmol, 63%). ^1^H NMR (400 MHz, DMSO-*d*_6_) δ 7.04 (td, *J* = 7.5, 1.7 Hz, 1H), 6.94 (dd, *J* = 7.5, 1.7 Hz, 1H), 6.88 (td, *J* = 7.3, 1.3 Hz, 1H), 6.77 (dd, *J* = 7.8, 1.3 Hz, 1H), 4.58 (q, *J* = 6.5 Hz, 1H), 2.13 (q, *J* = 7.6 Hz, 2H), 1.25 (d, *J* = 6.5 Hz, 3H), 1.10 (t, *J* = 7.6 Hz, 3H). ^13^C NMR (101 MHz, DMSO-*d*_6_) δ 158.9*, 140.9*, 127.2, 126.9*, 125.2, 124.4*, 122.8, 48.1*, 28.3, 25.5, 11.4. IR, v_max_/cm^−1^: 3234, 2979, 1658, 1576, 1487, 1451, 1398, 1299, 754. HRMS (ES TOF) calculated for (M + H)^+^ C_11_H_15_N_2_ 175.1235, found 175.1230 (−2.8 ppm).

*4-Methyl-2-propyl-3,4-dihydroquinazoline* (**3ad**):

Viscous glassy oil of amber color, *R_f_* 0.62 (EtOAc/EtOH/TEA, 32:8:1). Yield: 128 mg (0.68 mmol, 68%). ^1^H NMR (400 MHz, DMSO-*d*_6_) δ 7.04 (td, *J* = 7.5, 1.7 Hz, 1H), 6.94 (dd, *J* = 7.7, 1.7 Hz, 1H), 6.88 (td, *J* = 7.3, 1.3 Hz, 1H), 6.77 (dd, *J* = 7.8, 1.3 Hz, 1H), 4.58 (q, *J* = 6.4 Hz, 1H), 2.08 (td, *J* = 7.3, 1.7 Hz, 2H), 1.60 (q, *J* = 7.4 Hz, 2H), 1.24 (d, *J* = 6.4 Hz, 3H), 0.90 (t, *J* = 7.4 Hz, 3H). ^13^C NMR (101 MHz, Chloroform-*d*) δ 159.2, 137.7, 128.4, 125.5, 125.2, 124.6, 120.5, 49.1, 36.8, 25.9, 21.1, 13.7. IR, v_max_/cm^−1^: 3224, 2964, 1658, 1614, 1578, 1481, 1452, 1368, 1327, 1272, 751. HRMS (ES TOF) calculated for (M + H)^+^ C_12_H_17_N_2_ 189.1381, found 189.1386 (2.8 ppm).

*2-Butyl-4-methyl-3,4-dihydroquinazoline* (**3ae**):

Viscous glassy oil of amber color, *R_f_* 0.73 (EtOAc/EtOH/TEA, 32:8:1). Yield: 123 mg (0.61 mmol, 61%). ^1^H NMR (400 MHz, DMSO-*d*_6_) δ 7.04 (td, *J* = 7.5, 1.7 Hz, 1H), 6.94 (dd, *J* = 7.6, 1.7 Hz, 1H), 6.88 (td, *J* = 7.3, 1.3 Hz, 1H), 6.77 (dd, *J* = 7.8, 1.3 Hz, 1H), 4.58 (q, *J* = 6.4 Hz, 1H), 2.15–2.08 (m, 2H), 1.61–1.52 (m, 2H), 1.35–1.28 (m, 2H), 1.24 (d, *J* = 6.4 Hz, 3H), 0.89 (t, *J* = 7.4 Hz, 3H). ^13^C NMR (101 MHz, Chloroform-*d*) δ 159.2, 137.7, 128.4, 125.5, 125.2, 124.6, 120.5, 49.1, 36.8, 25.9, 21.1, 13.7. IR, v_max_/cm^−1^: 3297, 2959, 1655, 1616, 1578, 1481, 1455, 1375, 1272, 749. HRMS (ES TOF) calculated for (M + H)^+^ C_13_H_19_N_2_ 203.1543, found 203.1543 (0.0 ppm).

*4-Methyl-2-pentyl-3,4-dihydroquinazoline* (**3af**):

Viscous glassy oil of amber color, *Rf* 0.78 (EtOAc/EtOH/TEA, 32:8:1). Yield: 110 mg (0.51 mmol, 51%). ^1^H NMR (400 MHz, DMSO-*d*_6_) δ 7.04 (td, *J* = 7.5, 1.7 Hz, 1H), 6.94 (dd, *J* = 7.6, 1.7 Hz, 1H), 6.88 (td, *J* = 7.3, 1.3 Hz, 1H), 6.76 (d, *J* = 7.8 Hz, 1H), 4.57 (q, *J* = 6.4 Hz, 1H), 2.16–2.03 (m, 2H), 1.58 (p, *J* = 7.4 Hz, 2H), 1.30 (dp, *J* = 9.1, 5.2 Hz, 4H), 1.24 (d, *J* = 6.4 Hz, 3H), 0.87 (t, *J* = 6.9 Hz, 3H). ^13^C NMR (101 MHz, DMSO-*d*_6_) δ 157.6*, 141.5*, 127.4*, 127.3, 125.2, 123.0*, 122.8, 48.3*, 35.1, 30.9, 26.4, 25.6, 22.0, 14.0. IR, v_max_/cm^−1^: 3205, 2964, 1653, 1617, 1581, 1482, 1455, 1373, 1272, 752. HRMS (ES TOF) calculated for (M + H)^+^ C_14_H_21_N_2_ 217.1693, found 217.1699 (2.7 ppm).

*4-Methyl-2-phenyl-3,4-dihydroquinazoline* (**3ag**):

A light-yellow, glassy oil (Literature data: mp 70 °C [52]), *R_f_* 0.38 (EtOAc/EtOH, 4:1). Yield: 102 mg (0.46 mmol, 46%). ^1^H NMR (400 MHz, Chloroform-*d*) δ 7.81–7.72 (m, 2H), 7.45–7.33 (m, 3H), 7.18–7.08 (m, 2H), 7.03–6.98 (m, 1H), 6.95 (dd, *J* = 7.6, 1.4 Hz, 1H), 4.84 (q, *J* = 6.5 Hz, 1H), 1.46 (d, *J* = 6.5 Hz, 3H). ^13^C NMR (101 MHz, Chloroform-*d*) δ 154.1, 141.7, 135.8, 130.7, 128.8 (2C), 128.2, 126.7 (2C), 126.1, 125.2, 124.9, 49.5, 29.9, 25.8. IR, v_max_/cm^−1^: 3311, 2935, 1547, 1477, 1440, 1330, 1272, 1115, 1067, 1031. HRMS (ES TOF) calculated for (M + H)^+^ C_15_H_15_N_2_ 223.1228, found 223.1230 (0.9 ppm).

*6-Methyl-3,4-dihydroquinazoline* (**3ba**):

A light-yellow, viscous, glassy oil crystallizing over time, *R_f_* 0.23 (EtOAc/EtOH/TEA, 40:20:1). Yield: 102 mg (0.70 mmol, 70%). ^1^H NMR (400 MHz, Chloroform-*d*) δ 7.14 (s, 1H), 6.97–6.90 (m, 1H), 6.78 (d, *J* = 7.9 Hz, 1H), 6.68 (d, *J* = 1.9 Hz, 1H), 4.60 (s, 2H), 2.26 (s, 3H). ^13^C NMR (101 MHz, Chloroform-*d*) δ 147.1, 137.0*, 134.1, 129.0, 127.0, 120.8, 116.6, 44.2, 21.2. IR, v_max_/cm^−1^: 3186, 2983, 1747, 1660, 1624, 1597, 1373, 1318, 1265, 1243, 828. HRMS (ES TOF) calculated for (M + H)^+^ C_9_H_11_N_2_ 147.0914, found 147.0917 (2.1 ppm).

*2,6-Dimethyl-3,4-dihydroquinazoline* (**3bb**):

A light-yellow crystalline powder, *R_f_* 0.16 (EtOAc/EtOH/TEA, 40:20:1). Yield: 99 mg (0.62 mmol, 62%). ^1^H NMR (400 MHz, Chloroform-*d*) δ 6.96–6.91 (m, 1H), 6.80 (d, *J* = 8.0 Hz, 1H), 6.69 (t, *J* = 1.4 Hz, 1H), 4.58 (s, 2H), 2.25 (s, 3H), 2.01 (s, 3H). ^13^C NMR (101 MHz, Chloroform-*d*) δ 154.7*, 138.1*, 133.8, 128.7, 126.3, 119.6, 44.6, 22.7, 21.0. IR, v_max_/cm^−1^: 3215 2906, 1747, 1655, 1619, 1586, 1499, 1381, 1323, 1243, 826. HRMS (ES TOF) calculated for (M + H)^+^ C_10_H_13_N_2_ 161.1071, found 161.1073 (1.6 ppm).

*2-Ethyl-6-methyl-3,4-dihydroquinazoline* (**3bc**):

A light-yellow, viscous, glassy oil crystallizing over time, *R_f_* 0.31 (EtOAc/EtOH/TEA, 40:20:1). Yield: 134 mg (0.77 mmol, 77%). ^1^H NMR (400 MHz, DMSO-*d*_6_) δ 6.87–6.80 (m, 1H), 6.71–6.65 (m, 1H), 6.63 (d, *J* = 7.9 Hz, 1H), 4.40 (s, 2H), 2.17 (s, 3H), 2.10 (q, *J* = 7.6 Hz, 2H), 1.07 (t, *J* = 7.6 Hz, 3H). ^13^C NMR (101 MHz, DMSO-*d*_6_) δ 160.1, 153.3, 139.0*, 131.5, 127.79, 126.0, 119.7, 43.9*, 28.1, 20.5, 11.2. IR, v_max_/cm^−1^: 3258, 2993, 1653, 1622, 1496, 1373, 1270, 1238, 959, 824. HRMS (ES TOF) calculated for (M + H)^+^ C_11_H_15_N_2_ 175.1234, found 175.1230 (−2.4 ppm).

*6-Methyl-2-propyl-3,4-dihydroquinazoline* (**3bd**):

A light-yellow, viscous, glassy oil crystallizing over time, *R_f_* 0.47 (EtOAc/EtOH/TEA, 40:20:1). Yield: 162 mg (0.86 mmol, 86%). ^1^H NMR (400 MHz, Chloroform-*d*) δ 6.96–6.90 (m, 1H), 6.80 (d, *J* = 8.0 Hz, 1H), 6.72–6.65 (m, 1H), 4.57 (s, 2H), 2.25 (s, 3H), 2.22–2.18 (m, 2H), 1.70–1.64 (m, 2H), 0.97 (t, *J* = 7.4 Hz, 3H). ^13^C NMR (101 MHz, Chloroform-*d*) δ 157.9, 136.2, 134.1, 133.3, 128.3, 125.9, 119.5, 44.3, 38.1, 20.7, 20.5, 13.6. IR, v_max_/cm^−1^: 3253, 2964, 2930, 1747, 1655, 1622, 1586, 1508, 1489, 1378, 1320, 1241, 829. HRMS (ES TOF) calculated for (M + H)^+^ C_12_H_17_N_2_ 189.1390, found 189.1386 (−2.0 ppm).

*2-Butyl-6-methyl-3,4-dihydroquinazoline* (**3be**):

A light-yellow, viscous, glassy oil crystallizing over time, *R_f_* 0.55 (EtOAc/EtOH/TEA, 40:20:1). Yield: 162 mg (0.80 mmol, 80%). ^1^H NMR (400 MHz, Chloroform-*d*) δ 6.95–6.90 (m, 1H), 6.80 (d, *J* = 7.9 Hz, 1H), 6.69 (d, *J* = 1.9 Hz, 1H), 4.58 (s, 2H), 2.25 (s, 3H), 2.24–2.20 (m, 2H), 1.65–1.59 (m, 2H), 1.42–1.36 (m, 2H), 0.92 (t, *J* = 7.3 Hz, 3H). ^13^C NMR (101 MHz, Chloroform-*d*) δ 159.9, 138.4*, 133.3, 129.9, 128.6, 126.3, 119.8, 44.7, 36.4, 29.6, 22.1, 21.0, 14.0. IR, v_max_/cm^−1^: 3263, 3195, 2964, 2935, 1742, 1653, 1622, 1586, 1510, 1489, 1378, 1322, 1270, 1241, 822. HRMS (ES TOF) calculated for (M + H)^+^ C_13_H_19_N_2_ 203.1539, found 203.1543 (1.9 ppm).

*6-Methyl-2-pentyl-3,4-dihydroquinazoline* (**3bf**):

A light-yellow, viscous, glassy oil, *Rf* 0.66 (EtOAc/EtOH/TEA, 40:20:1). Yield: 169 mg (0.78 mmol, 78%). ^1^H NMR (400 MHz, Chloroform-*d*) δ 6.93 (dd, *J* = 7.8, 2.2 Hz, 1H), 6.80 (d, *J* = 8.1 Hz, 1H), 6.69 (d, *J* = 1.9 Hz, 1H), 4.58 (s, 2H), 2.26–2.25 (m, 3H), 2.24–2.20 (m, 2H), 1.67–1.61 (m, 2H), 1.33 (dq, *J* = 6.6, 3.0 Hz, 4H), 0.91–0.86 (m, 3H). ^13^C NMR (101 MHz, Chloroform-*d*) δ 158.4*, 135.2*, 133.7, 129.9, 128.6, 126.3, 119.8, 44.5, 36.6, 31.7, 27.2, 22.6, 21.0, 14.1. IR, v_max_/cm^−1^: 3292, 3195, 2964, 2939, 1747, 1655, 1588, 1511, 1492, 1381, 1323, 1251, 821. HRMS (ES TOF) calculated for (M + H)^+^ C_14_H_21_N_2_ 217.1702, found 217.1699 (−1.1 ppm).

*3,4-Dihydroquinazoline* (**3ca**):

Off-white solid, mp 123–125 °C (Literature data: mp 123–126.5 °C [53]), *R_f_* 0.37 (EtOAc/EtOH/TEA, 12:6:1). Yield: 107 mg (0.81 mmol, 81%). ^1^H NMR (400 MHz, Chloroform-*d*) δ 7.15 (s, 1H), 7.06 (td, *J* = 7.6, 1.5 Hz, 1H), 6.92 (td, *J* = 7.5, 1.2 Hz, 1H), 6.84–6.74 (m, 2H), 5.88 (s, 1H), 4.58 (s, 2H). ^13^C NMR (101 MHz, Chloroform-*d*) δ 147.2, 139.0, 128.0, 126.0, 124.6, 120.1, 119.7, 44.0. IR, v_max_/cm^−1^: 3344, 3234, 3041, 2872, 1662, 1493, 1375, 1146, 1077, 913. HRMS (ES TOF) calculated for (M + H)^+^ C_8_H_9_N_2_ 133.0756, found 133.0760 (2.8 ppm).

*2-Methyl-3,4-dihydroquinazoline* (**3cb**):

Yellow gummy oil, *Rf.* 0.40 (EtOAc/EtOH/TEA 12:6:1). Yield: 105 mg (0.72 mmol, 72%). ^1^H NMR (400 MHz, Chloroform-*d*) δ 7.05 (t, *J* = 1.0 Hz, 1H), 6.90 (td, *J* = 7.4, 1.3 Hz, 1H), 6.81 (ddd, *J* = 7.0, 5.6, 1.3 Hz, 2H), 5.81 (s, 1H), 4.55 (s, 2H), 1.97 (s, 3H). ^13^C NMR (101 MHz, Chloroform-*d*) δ 155.4, 140.3, 128.0, 125.6, 124.1, 120.0, 118.8, 44.5, 22.1. IR, v_max_/cm^−1^: 3209, 3017, 2877, 2829, 1660, 1587, 1481, 1334, 1272. HRMS (ES TOF) calculated for (M + H)^+^ C_9_H_11_N_2_ 147.0917, found 147.0917 (−0.5 ppm).

*2-Ethyl-3,4-dihydroquinazoline* (**3cc**):

Colorless solid, mp 101–103 °C (Literature data mp 103–104 °C (benzene, petroleum ether) [54]), *R_f_* 0.43 (EtOAc/EtOH/TEA, 10:4:1). Yield: 112 mg (0.70 mmol, 70%). ^1^H NMR (400 MHz, Chloroform-*d*) δ 7.17–7.09 (m, 1H), 6.98 (td, *J* = 7.4, 1.2 Hz, 1H), 6.90 (dd, *J* = 7.8, 1.4 Hz, 2H), 5.57 (s, 1H), 4.64 (s, 2H), 2.30 (q, *J* = 7.6 Hz, 2H), 1.24 (t, *J* = 7.6 Hz, 3H). ^13^C NMR (101 MHz, Chloroform-*d*) δ 161.3, 138.3, 127.9, 125.6, 123.8, 119.7, 119.6, 43.4, 29.2, 11.4. IR, v_max_/cm^−1^: 2922, 2848, 1975, 1944, 1836, 1766, 1674, 1604, 1457, 954. HRMS (ES TOF) calculated for (M + H)^+^ C_10_H_13_N_2_ 161.1068, found 161.1073 (3.0 ppm).

*2-Propyl-3,4-dihydroquinazoline* (**3cd**):

Yellow gummy oil, *Rf* 0.43 (EtOAc/EtOH, 5:1). Yield: 113 mg (0.65 mmol, 65%). ^1^H NMR (400 MHz, Chloroform-*d*)) δ 7.15 (td, *J* = 7.6, 1.5 Hz, 1H), 6.99 (td, *J* = 7.4, 1.3 Hz, 1H), 6.90 (d, *J* = 7.3 Hz, 2H), 4.65 (s, 2H), 4.11 (s, 1H), 2.31–2.21 (m, 2H), 1.77–1.65 (m, 2H), 1.01 (t, *J* = 7.3 Hz, 3H). ^13^C NMR (101 MHz, Chloroform-*d*) δ 158.87, 140.89, 128.40, 126.05, 124.40, 120.47, 120.16, 45.11, 38.65, 21.14, 14.24. IR, v_max_/cm^−1^: 2607, 2327, 1648, 1585, 1498, 1397, 1327, 1245, 1057, 886. HRMS (ES TOF) calculated for (M + H)^+^ C_11_H_15_N_2_ 175.1225, found 175.1230 (2.9 ppm).

*2-Butyl-3,4-dihydroquinazoline* (**3ce**):

Yellow gummy oil, *R_f_* 0.50 (EtOAc/EtOH, 5:2). Yield: 105 mg (0.56 mmol, 56%). ^1^H NMR (400 MHz, DMSO) δ 7.04 (ddd, *J* = 7.8, 5.6, 3.3 Hz, 1H), 6.91–6.83 (m, 2H), 6.75 (d, *J* = 7.8 Hz, 1H), 4.44 (s, 2H), 2.17–2.04 (m, 2H), 1.55 (tt, *J* = 8.9, 6.8 Hz, 2H), 1.40–1.24 (m, 2H), 0.88 (t, *J* = 7.4 Hz, 3H). ^13^C NMR (101 MHz, DMSO-*d*_6_) δ 159.4, 140.5, 128.0, 126.1, 123.6, 119.8, 118.7, 44.3, 34.6, 29.1, 22.2, 14.2. IR, v_max_/cm^−1^: 3195, 3002, 2853, 2356, 1773, 1580, 1436, 1149, 1055. HRMS (ES TOF) calculated for (M + H)^+^ C_12_H_17_N_2_ 189.1386, found 189.1386 (0.1 ppm).

*2-Pentyl-3,4-dihydroquinazoline* (**3cf**):

Colorless gummy oil, *R_f_* 0.67 (EtOAc/EtOH, 5:2). Yield: 101 mg (0.50 mmol, 50%). ^1^H NMR (400 MHz, DMSO) δ 7.04 (ddd, *J* = 8.2, 5.6, 3.2 Hz, 1H), 6.87 (d, *J* = 3.3 Hz, 2H), 6.74 (d, *J* = 7.8 Hz, 1H), 4.43 (s, 2H), 2.15–2.04 (m, 2H), 1.57 (p, *J* = 7.4 Hz, 2H), 1.36–1.17 (m, 4H), 0.87 (t, *J* = 6.8 Hz, 3H). ^13^C NMR (101 MHz, DMSO-*d*_6_) δ 158.8, 141.4, 127.8, 125.9, 123.2, 120.1, 119.0, 44.6, 35.3, 31.4, 26.6, 22.3, 14.3. IR, v_max_/cm^−1^: 3195, 3026, 2858, 1640, 1433, 1151, 1045, 995, 896. HRMS (ES TOF) calculated for (M + H)^+^ C_13_H_19_N_2_ 203.1540, found 203.1543 (1.5 ppm).

*2-Phenyl-3,4-dihydroquinazoline* (**3cg**):

Yield: 114 mg (0.55 mmol, 55%) off-white solid, mp: 133–135 °C (Literature data [48]), R*_f_* 0.45 (dichloromethane/MeOH, 95:5). ^1^H NMR (400 MHz, Chloroform-*d*) δ 7.97 (d, *J* = 7.6 Hz, 2H), 7.53 (d, *J* = 7.9 Hz, 1H), 7.34 (t, *J* = 7.3 Hz, 1H), 7.30–7.26 (m, 2H), 7.20 (t, *J* = 7.6 Hz, 1H), 7.13 (d, *J* = 7.4 Hz, 1H), 6.94 (d, *J* = 7.5 Hz, 1H), 4.70 (s, 2H).

*6-Bromo-2-ethyl-4-methyl-3,4-dihydroquinazoline* (**3dc**):

Light yellow glassy oil, *R_f_* 0.20 (EtOAc/EtOH, 4:1). Yield: 186 mg (0.74 mmol, 74%). ^1^H NMR (400 MHz, Chloroform-*d*) δ 7.23 (dd, *J* = 8.4, 2.3 Hz, 1H), 7.04 (d, *J* = 2.3 Hz, 1H), 6.89–6.85 (m, 1H), 5.07 (br. s, 1H), 4.68 (q, *J* = 6.5 Hz, 1H), 2.27 (q, *J* = 7.6 Hz, 2H), 1.39 (d, *J* = 6.5 Hz, 3H), 1.21 (t, *J* = 7.7 Hz, 3H). ^13^C NMR (101 MHz, Chloroform-*d*) δ 159.4, 140.5, 131.0, 128.1, 127.6, 124.1, 116.4, 48.7, 29.7, 25.8, 11.6. IR, v_max_/cm^−1^: 3287, 2973, 1607, 1580, 1540, 1472, 1440, 1404, 1294, 1262, 1064,879. HRMS (ES TOF) calculated for (M + H)^+^ C_11_H_14_BrN_2_ 253.0331, found 253.0335 (1.7 ppm).

## 4. Conclusions

In conclusion, a new protocol was developed, allowing for the assembly of 3,4-dihydroquinazolines from readily available 2-(aminomethyl)anilines and electrophilically activated nitroalkanes. Typically, reactions proceeding via nucleophilic attacks of nitronates with nucleophilic aniline moieties can be performed routinely. However, the featured transformation, involving the reaction of aliphatic amine groups, required careful optimization of the reaction conditions. It was found that switching to a mixture of 87% PPA and H_3_PO_3_ as a reaction medium allowed the transformation to be carried out efficiently, obtaining adequate to high yields of 3,4-dihydroquinazolines from a range of linear nitroalkanes (from nitromethane to 1-nitrohexane). Reaction with α-nitroacetophenone allowed for efficient preparation of phenyl-substituted derivatives. Evidently, the described method largely depends on availability and stability of the corresponding starting materials. Since nitroalkanes are activated at quite high temperatures and are used in the reaction as surrogates for much more available and reactive carboxylic acid derivatives, it is hard to expect that the method as is would be strongly competitive. This reaction might become invaluable, however, as a part of multi-step cascade transformations involving PPA as an “intelligent” reaction medium, which are being developed lately in our laboratories. 

## Figures and Tables

**Table 1:**
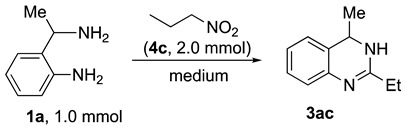
Optimization of reaction conditions for annulation.

	**Medium**	**t, °C**	**Time, h**	**Yield, % ^1^**
1	H_3_PO_4_ 86%, 2 g	110	4	0
2	H_3_PO_4_ 86%, 2 g	120	4	0
3	H_3_PO_4_ 86%, 1 g PPA 87%, 1 g	120	4	0
4	PPA 87%, 2 g	120	4	0
5	PPA 87%, 1 g; H_3_PO_3_, 1 g	130	4	9
6	PPA 87%, 1 g; H_3_PO_3_, 1 g	150	2	40
7	PPA 87%, 1 g; H_3_PO_3_, 1 g	160	2	63 ^2^
8	H_3_PO_3_, 2 g	160	2	51 ^2^

^1^ NMR yields are provided unless specified otherwise. ^2^ Isolated yield of purified product is reported.

## Data Availability

Supporting Information data include NMR spectral charts and are available.
